# Prevalence of Latent and Active Tuberculosis among Dairy Farm Workers Exposed to Cattle Infected by *Mycobacterium bovis*

**DOI:** 10.1371/journal.pntd.0002177

**Published:** 2013-04-25

**Authors:** Pedro Torres-Gonzalez, Orbelin Soberanis-Ramos, Areli Martinez-Gamboa, Barbara Chavez-Mazari, Ma Teresa Barrios-Herrera, Martha Torres-Rojas, Luis Pablo Cruz-Hervert, Lourdes Garcia-Garcia, Mahavir Singh, Adrian Gonzalez-Aguirre, Alfredo Ponce de Leon-Garduño, José Sifuentes-Osornio, Miriam Bobadilla-del-Valle

**Affiliations:** 1 Laboratory of Clinical Microbiology, Instituto Nacional de Ciencias Medicas y Nutricion Salvador Zubiran, Mexico City, Mexico; 2 Department of Public Health, Facultad de Medicina Veterinaria y Zootecnia, Universidad Nacional Autónoma de Mexico, Mexico City, Mexico; 3 Department of Microbiology, Instituto Nacional de Enfermedades Respiratorias Ismael Cosio Villegas, Mexico City, Mexico; 4 Centro de Investigacion sobre Enfermedades Infecciosas, Instituto Nacional de Salud Publica, Cuernavaca Morelos, Mexico; 5 Lionex GmbH, Braunschweig, Germany; 6 Department of Radiology, Instituto Nacional de Ciencias Medicas y Nutricion Salvador Zubiran, Mexico City, Mexico; 7 Department of Medicine, Instituto Nacional de Ciencias Médicas y Nutrición Salvador Zubirán, Mexico City, Mexico; University of Tennessee, United States of America

## Abstract

**Background:**

Human tuberculosis caused by *M. bovis* is a zoonosis presently considered sporadic in developed countries, but remains a poorly studied problem in low and middle resource countries. The disease in humans is mainly attributed to unpasteurized dairy products consumption. However, transmission due to exposure of humans to infected animals has been also recognized. The prevalence of tuberculosis infection and associated risk factors have been insufficiently characterized among dairy farm workers (DFW) exposed in settings with poor control of bovine tuberculosis.

**Methodology/Principal Findings:**

Tuberculin skin test (TST) and Interferon-gamma release assay (IGRA) were administered to 311 dairy farm and abattoir workers and their household contacts linked to a dairy production and livestock facility in Mexico. Sputa of individuals with respiratory symptoms and samples from routine cattle necropsies were cultured for *M. bovis* and resulting spoligotypes were compared. The overall prevalence of latent tuberculosis infection (LTBI) was 76.2% (95% CI, 71.4–80.9%) by TST and 58.5% (95% CI, 53.0–64.0%) by IGRA. Occupational exposure was associated to TST (OR 2.72; 95% CI, 1.31–5.64) and IGRA (OR 2.38; 95% CI, 1.31–4.30) adjusting for relevant variables. Two subjects were diagnosed with pulmonary tuberculosis, both caused by *M. bovis*. In one case, the spoligotype was identical to a strain isolated from bovines.

**Conclusions:**

We documented a high prevalence of latent and pulmonary TB among workers exposed to cattle infected with *M. bovis*, and increased risk among those occupationally exposed in non-ventilated spaces. Interspecies transmission is frequent and represents an occupational hazard in this setting.

## Author Summary

*Mycobacterium tuberculosis* complex causes tuberculosis in humans and other mammals. The complex includes *M. bovis*, which causes bovine tuberculosis. The main route of transmission of this zoonosis is the consumption of unpasteurized dairy products. Nevertheless, exposure to infected cattle while performing husbandry and farm activities may cause disease as well. In this study we were able to demonstrate: 1) A high prevalence of tuberculosis asymptomatic infection (latent tuberculosis) among workers exposed to infected cattle; 2) A higher probability of infection among individuals who are occupationally exposed in closed spaces; and 3) Cattle to human transmission confirmed by molecular methods (spoligotyping). We conclude that occupational exposure is frequent, and therefore strict prevention and control measures are required in these settings.

## Introduction

The most frequent etiologic agent of tuberculosis (TB) in humans is *Mycobacterium tuberculosis*, followed by *M. bovis*, that causes bovine tuberculosis [1]. The zoonosis caused by *M. bovis* in developed countries is now considered a sporadic disease since control of the disease in cattle was achieved and pasteurization of dairy products became extensively practiced [2], [3]. Nevertheless, an increase in the number cases of TB caused by *M. bovis* in some regions of the United States of America has recently been reported and it has been attributed to migrant populations from low and middle resource countries (specially from Mexico), and in most cases, associated to consumption of unpasteurized dairy products [4], [5], [6]. Moreover, we have recently noted an increase in TB cases due *M. bovis* in a tertiary-care hospital in Mexico, accounting for the 22.3% of the TB isolates from 2000–2007 (Franco-Cendejas R, et al. *Mycobacterium bovis* infections in a tertiary-care centre in Mexico: A case-control study. 20^th^ ECCMID. Vienna, Austria. April 10–13, 2010. Poster 108).

Most of the human cases of contagion by close contact with infected animals, occurring among veterinarians, zoo workers, hunters, abattoir workers and dairy farm workers (DFW) have been reported in developed countries [7], [8], [9], [10], [11], but the situation in low and middle resource countries, where the control of the disease in cattle is poor and the human-cattle interface is more likely to occur, remains poorly documented [12]. Because of insufficient information, the World Health Organization recommended the collection of epidemiological data about human tuberculosis due to *M. bovis* specially in these regions [13].

Previous medical literature examining this zoonosis shows important limitations. Most studies have focused on active disease, and only a few have reported the prevalence of latent tuberculosis [10], [11], [14]. Until the past decade, the only diagnostic test available to identify LTBI was the tuberculin skin test (TST). Presently, interferon-γ release assays (IGRA) are considered an interchangeable or complementary test to TST by international guidelines [15], [16]. To our knowledge, these tests have never been used to detect LTBI in DFW exposed to infected cattle.

The main objective of our study was to determine the prevalence of active tuberculosis and LTBI among DFW with different degrees of exposure to cattle, the risk associated to occupational exposure and the occurrence of interspecies transmission.

## Methods

### Description of study area

Dairy production facility: The study was performed in a dairy production and livestock facility located in a municipality of the state of Hidalgo, Mexico. This facility was composed by 126 cowsheds in dairy production, with an average cattle population of 29,000. Inside most of the cowsheds, improvised housing was provided to workers and their families in small rooms near to the areas where farming activities occurred.

Abattoir house: We also included workers from an abattoir house in the same municipality that processed cattle from the studied dairy farm facility.

Design and study period: A cross-sectional, comparative study was conducted during 2009–2011.

### Study population

We requested authorization from owners of the 126 cowsheds within the facility to invite their employees to the study. On those facilities where authorization was granted, all the abattoir and dairy production workers and their household contacts living inside the improvised housing in cowsheds, older than 15 years were invited to participate.

### Procedures

A standardized questionnaire (investigating exposure to TB cases, consumption of unpasteurized dairy products, BCG vaccination status as well as other socio-demographic and health conditions), full medical exam, TST and IGRA were administered to all consenting participants. Active TB was investigated on individuals with respiratory or systemic symptoms by AFB smear and culture of appropriate sputum samples and chest X-ray. Fasting glucose, complete blood count, urine exam and chest X-ray were also performed.

### Tuberculin skin test and Interferon-γ release assay

TST was performed by the Mantoux method administering 5 IU (0.1 ml) of PPD (Tubersol Sanofi-Pasteur, Toronto, CA) in the volar surface of the forearm. After 48–72 hr, the reading was performed by previously trained personal using a caliper to measure the induration and the result was registered in millimeters and determined positive using a 10 mm cut-off. In those cases with a negative result a booster test was administered after three weeks. Venous blood was drawn to determine interferon gamma response to ESAT-6 and CFP-10 antigens by ELISPOT (Lionex Diagnostics & Therapeutics GmbH, Braunschweig, Germany). The test was determined positive if more than 6 spots were observed.

### Tuberculosis burden in bovine population

To determine the bovine tuberculosis burden and circulating strains in the cattle, a parallel surveillance was conducted in animals during the same period. As a regular practice in the facility, the cowshed owners are requested to perform a necropsy study to cattle that dies within the facility. Using standardized procedures, a veterinarian visually inspected lymph nodes, lungs and other organs for lesions suggestive of tuberculosis. Data from all necropsy reports were obtained during the study period and tuberculosis macroscopic diagnosis were recorded. Tissue samples were obtained for *M. bovis* culture and spoligotyping whenever possible. Slaughtered animals were not formally inspected and no records as to the macroscopic findings were available or informed to us.

### Microbiological and molecular methods

In those subjects who reported respiratory symptoms for more than 2 weeks, 3 sputa samples were collected and Ziehl-Neelsen stains were examined microscopically for the presence of acid-fast bacilli (AFB).

The samples from humans and animals were decontaminated and digested to increase the positivity yield, and were inoculated in solid culture media (Lowestein-Jensen and Stonebrink) and MGIT tubes (Becton-Dickinson, Sparks, MA, USA) according to the manufacturer's specifications. Positive cultures were identified as *M. bovis* by conventional biochemical tests, and DNA probing (Accuprobe, GEN-PROBE, San Diego, CA). DR region was amplified by polymerase chain reaction (PCR) and then analyzed according to the spoligotyping protocol, as described elsewhere [17], *M. tuberculosis* H37Rv and *M. bovis* BCGP3 were used as controls. Data from spoligotyping of the strains was introduced in an international database (www.mbovis.org).

### Statistical analysis

The following exposure groups were formed according to type of activity, duration and conditions of exposure to cattle; high exposure: individuals in direct contact with livestock working in closed spaces (abattoir workers; veterinary personnel performing cattle necropsies, foremen and milkers), medium exposure: participants working in open spaces in direct contact with cattle (tractor operators, breeders, those in charge of providing food to cattle, veterinary personal, and maintenance technicians) and household contacts living in the cowshed; and low exposure: workers without direct contact with livestock (owners of the cowshed, administrative clerks, and people involved in commercial activities) (Table 1).

**Table 1:** Categories of exposure to cattle among study population according to duration and conditions of exposure to cattle.

Categories of exposure	Duration and conditions of exposure to cattle	Type of activity/exposure group
High	Direct contact with livestock in closed spaces	Abattoir workers
		Veterinary personal performing cattle necropsies
		Foremen
		Milkers
Medium	Direct contact with livestock in open spaces	Tractor operators
		Breeders
		Feeders
		Other veterinary personal
		Maintenance technicians
		Household contacts living in the cowshed
Low	No direct contact with livestock	Owners of the cowshed
		Administrative clerks
		People involved in commercial activities

Using bivariate and multivariate analyses (unconditional logistic regression) we tested the association between exposure groups and other relevant variables with LTBI (as determined by TST or IGRA). Three combinations were additionally analyzed: IGRA and TST positive compared with any other combination of test results, IGRA or TST positive compared with IGRA and TST negative results and IGRA and TST positive compared with IGRA and TST negative results. Variables with p<0.20 in the bivariate analysis and biological plausibility were included in multivariate models. We estimated the odds ratios (OR) and 95 percent confidence intervals (CI), and identified the covariates that were independently associated with each outcome. Statistical analysis was performed using STATA 11.0 software (StataCorp, College Station, Tx).

### Ethics

The aims of the study were communicated to the participants and a written informed consent form was signed before the inclusion to the study. Whenever the subjects were minors the informed consent was given by the parents or mentors. Respecting animal population the owners of the cattle were informed of the purpose of the study and their permission was given for data use and culture recollection during routine necropsy studies. The protocol was reviewed and approved by the Salvador Zubiran National Institute of Medical Sciences and Nutrition ethics committee (approval reference 234). All participants with abnormal results were referred for appropriate treatment. All contacts of pulmonary TB cases were studied as specified in the national regulations, although excluded from analysis if they lived outside the facility.

## Results

### Animal population

During the study period 1561/29,000 cows died within the facility. Necropsy was performed on 718/1561 of dead cattle. Macroscopic diagnosis of TB was made in 247/718 necropsies; of these, mycobacterial cultures were performed on 163/247 and *M. bovis* was identified on 154 instances (Table 2).

**Table 2:** Cattle infected with *Mycobacterium bovis* in dairy production and livestock facility in Mexico.

	No.(%)
Dead animals during study period	1561/29,000 (5.3)
Routine necropsies performed	718/1561 (45.9)
TB suggestive lesions in routine necropsies	247/718 (34.4)
Mycobacteria cultures performed	163/247 (65.9)
Isolation of *M. bovis* in cultures	154/163 (94.4)
Culture confirmed prevalence of bovine TB	154/1561 (9.8)

### Human population

**Figure 1:**
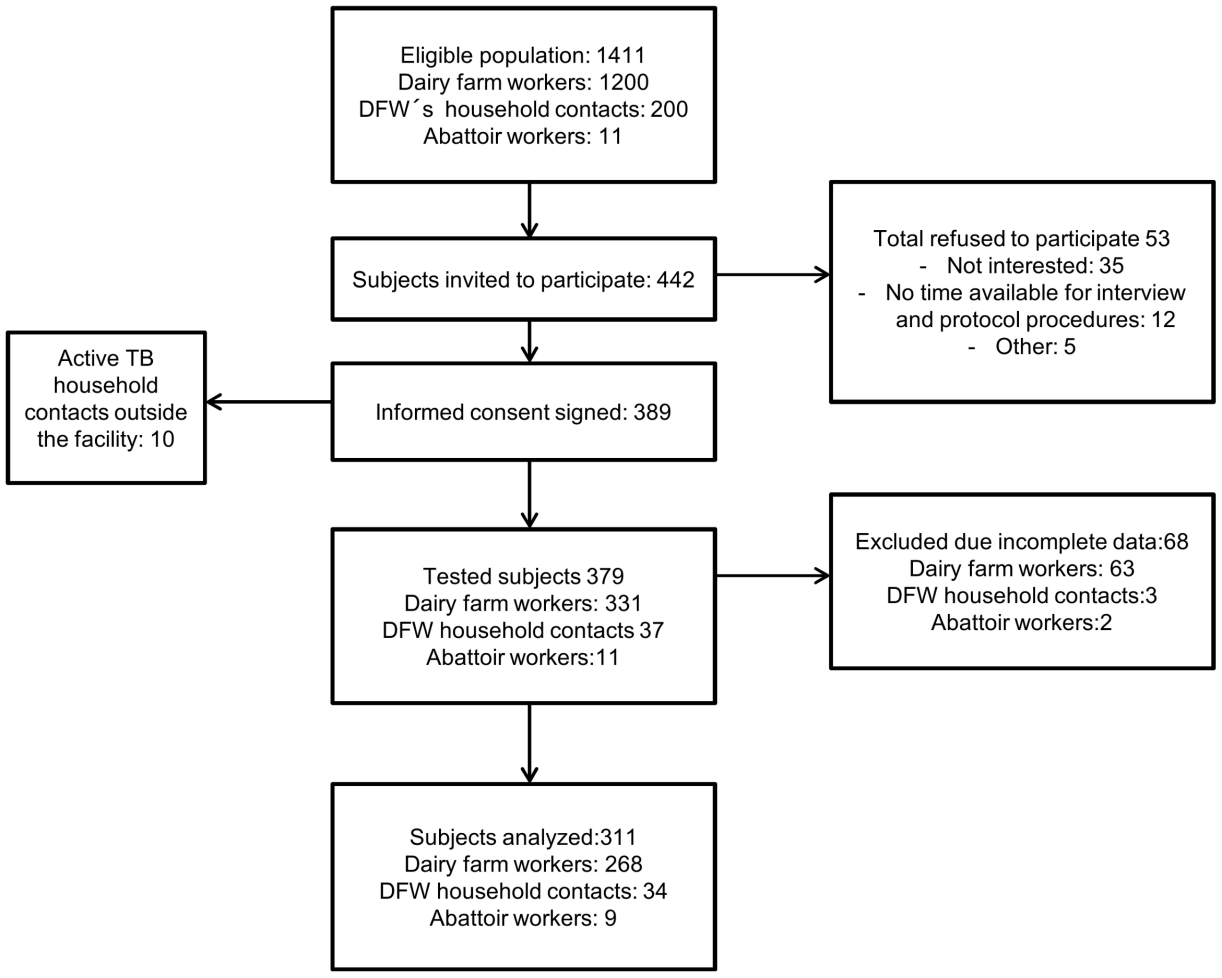
Flow chart of enrollment.

Estimated eligible population was comprised of 1,411 persons. Four hundred and forty two subjects from 56 different cowsheds were invited to participate, 389 subjects signed informed consent; 68 subjects were excluded of the study because of incomplete study procedures (TST or IGRA not performed) or residence outside the facility (10 household contacts). Data on 311 subjects were analyzed. (Fig. 1)

Median age of the subjects was 36 years (IQR 27–45), 78.0% were male. The median time living or working in the dairy facility was 132 months (IQR 72–240). One hundred and eighty eight subjects (60.4%) lived inside the cowshed at the moment of the study. The most frequently reported activities were milker (22.3%), followed by veterinarians (12.3%), diverse activities with no contact with cattle (12.3%), and feeders (11.0%). Household contacts represented 11.0% of study population. Abattoir workers accounted for 2.5%. The median time of daily exposure to cattle was 6 h (IQR 0–8). According to the level of exposure, 33.9% (105/309), 46.9% (145/309) and 19% (59/309) of the subjects were assigned to the high, medium and low exposure groups, respectively. (Table 3)

**Table 3:** Demographic and occupational characteristics of a study population (n = 311) linked to a dairy production and livestock facility in Mexico.

Characteristic	No.(%)
	Median (IQR)
Age	36 (27–45)
Men	244/311 (78.0)
Illiteracy	33/308 (10.1)
<6 year of formal education	137/310 (43.3)
Birth in a highly endemic area	104/311 (33.4)
Living inside a cowshed	188/311 (60.4)
Months working/living at daily facility	132 (72–240)
Group of exposure	
High	105/309 (33.9)
Medium	145/309 (46.9)
Low	59/309 (17.3)
Daily hours of contact with cattle	6 (0–8)
BCG scar	265/311 (85.2)
Previous contact with TB case	31/308 (10.0)
Consumption of unpasteurized dairy products	93/310 (30.0)
Previous TB diagnosis	4/311 (1.2)

IQR = interquartile range.

### Tuberculosis related characteristics

Eighty-five percent (265/311) of subjects had a BCG scar, 9.9% (31/311) informed previous close contact with a TB case, and 30.0% (93/310) informed consumption of unpasteurized dairy products. Fourteen per cent (37/251) of the chest X rays showed TB scars. Four subjects reported having been previously diagnosed with TB (1.29%) (Table 3).

### Tuberculin skin test

The median induration of the TST in the population was 14 mm (IQR 10–20), the prevalence of LTBI diagnosed by TST was 76.2% (95% CI, 71.4–80.9%). Reactivity was 86.6%, 70.3% and 71.1% among the high, medium and low exposure groups, respectively. Statistical difference was found between reactivity of the high exposure group when compared with medium and low exposure groups. For the rest of the analysis, activities comprised in the high exposure group were considered high exposure activities.

Thirty nine percent of the TST positive (91/235) subjects performed high exposure activities while 19% (14/74) of the TST negative subjects (p = 0.002) belonged to this group. Twenty seven per cent (64/236) of the TST positive subjects had history of unpasteurized products consumption compared with 39% (29/74) of the TST negative (p = 0.04). Sixty one percent (145/236) of the TST positive subjects reported more than 4 hours of daily contact with cattle while 49% (36/74) of the TST negative subjects reported that intensity of contact (p = 0.051). No other differences were found between other TB related characteristics and the TST positivity (Table 4). Multivariate analysis revealed association between the high exposure group and TST reactivity (OR 2.72; 95% CI 1.31–5.64, p = 0.003) adjusting by age, gender and other TB related co-variables (Table 5).

**Table 4:** Characteristics associated to tuberculosis skin test and interferon-γ release assay.

		TST		IGRA	
	Total (n = 311)	Positive	Negative	Positive	Negative
	No.(%)	No.(%)	No.(%)	No.(%)	No.(%)
Age in years, Median (S.D.)	36.9 (11.8)	36.8 (12.1)	37.1 (11.4)	36.8 (11.5)	37.5 (12.9)
Male	244/311 (78.0)	190/237 (80.0)	54/74 (73.0)	147/182 (81.0)	97/129 (75.0)
Birth in a highly endemic area	104/311 (33.0)	84/237 (35.0)	20/74 (27.0)	67/182 (37.0)	37/129 (29.0)
<6 y of formal education	137/310 (44.0)	106/236 (45.0)	31/74 (42.0)	80/182 (44.0)	57/128 (45.0)
>4 h of daily contact w/cattle	181/310 (58.0)	145/236 (61.0)*	36/74 (49.0)	107/182 (59.0)	74/128 (58.0)
>1 y of stay in the facility	283/309 (92.0)	214/236 (91.0)	69/73 (95.0)	171/181 (94.0)*	112/128 (88.0)
High exposure activity	105/309 (34.0)	91/235 (39.0)*	14/74 (19.0)	74/181 (41.0)*	31/128 (24.0)
Unpasteurized dairy products consumption	93/310 (30.0)	64/236 (27.0)*	29/74 (39.0)	56/181 (31.0)	37/129 (29.0)
BCG scar	265/301 (88.0)	203/229 (89.0)	62/72 (86.0)	154/175 (88.0)	111/126 (88.0)
Previous contact with TB cases	31/308 (10.0)	21/234 (9.0)	10/74 (14.0)	18/180 (10.0)	13/128 (10.0)

* p<0.05.

**Table 5:** Characteristics associated to tuberculosis skin test and interferon-γ release assay by multivariate analysis.

	TST	IGRA	TST+ & IGRA+ *vs* any other combination	TST+&/or IGRA+ *vs* TST−&IGRA−	TST+& IGRA+ *vs* TST− & IGRA
	OR (95%CI)	OR (95%CI)	OR (95%CI)	OR (95%CI)	OR (95%CI)
Age	1.00 (0.97–1.02)	0.99 (0.97–1.01)	0.99 (0.97–1.01)	0.99 (0.96–1.02)	0.99 (0.96–1.03)
Male	0.93 (0.43–1.99)	1.54 (0.78–3.05)	1.40 (0.71–2.75)	1.10 (0.43–2.77)	1.35 (0.49–3.68)
>4 h of daily contact w/cattle	1.20 (0.61–2.36)	0.61 (0.33–1.12)	0.70 (0.39–1.28)	1.02 (0.44–2.34)	0.85 (0.34–2.08)
>1 y of stay in the facility	0.57 (0.17–1.83)	2.70 (1.1–6.61)*	1.93 (0.78–4.79)	1.00 (0.26–3.83)	1.19 (0.27–5.20)
High exposure activity	2.72 (1.31–5.64)*	2.38 (1.31–4.30)*	2.49 (1.40–4.42)*	4.67 (1.62–13.47)*	6.09 (2.04–18.23)**
Unpasteurized dairy products consumption	0.49 (0.27–0.91)*	0.90 (0.52–1.54)	0.82 (0.48–1.40)	0.40 (0.19–0.85)*	0.40 (0.17–0.91)*
BCG scar	1.38 (0.6–3.17)	1.10 (0.52–2.33)	1.13 (0.54–2.35)	1.59 (0.57–4.41)	1.38 (0.46–4.09)
Previous contact with TB cases	0.68 (0.28–1.63)	1.10 (0.48–2.52)	1.13 (0.50–2.55)	0.58 (0.21–1.57)	0.68 (0.23–1.98)

* p<.05;

** p<.001.

### Interferon-γ release-assays

The prevalence of LTBI diagnosed by IGRA was 58.5% (95% CI, 53.0–64.0%). A positive test was found in 70.4%, 48.9% and 61.2% of individuals assigned to high, medium and low exposure groups respectively. As with TST results, statistical difference was found between positivity of the high exposure group when compared with medium and low exposure groups. For the rest of the analysis, activities comprised in the high exposure group were considered high exposure activities.

Ninety four percent (171/181) of the IGRA positive and 88.0% (112/128) of IGRA negative subjects had a length of stay at the facility longer than 1 year (p = 0.03). Forty one percent (74/181) of the IGRA positive and 24.0% (31/128) of the IGRA negative subjects performed high exposure activities (p = 0.002). No differences were found between other TB related characteristics and the IGRA positivity. (Table 4)

The high exposure group was associated with a positive result in the IGRA test (OR 2.38; 95% CI 1.31–4.30, p = 0.003) after adjusting by age, gender and other TB related co-variables. (Table 5)

### TST+ & IGRA+ vs. any other test result combination

Forty four percent (65/148) of the TST+ & IGRA+ subjects and 25% (40/161) of the subjects with any other combination performed high exposure activities (p<0.001). No other characteristic was found statistically different (Table 6). Multivariate analyses revealed association of this result with high exposure activities (OR 2.49 95% CI 1.40–4.42) (Table 5).

**Table 6:** Combined tests results of tuberculosis skin test and interferon-γ release assay.

		TST+ & IGRA+ *vs* any other combination		TST+&/or IGRA+ *vs* TST−&IGRA−		TST+& IGRA+ *vs* TST− & IGRA−		
	Total (n = 311)	Positive	Negative	Positive	Negative	Total (n = 190)	Positive	Negative
	No.(Col%)	No.(Col%)	No.(Col%)	No.(Col%)	No.(Col%)	No.(Col%)	No.(Col%)	No.(Col%)
Age in years, Median (SD)	36.9 (11.8)	36.8 (11.5)	37.0 (12.1)	36.8(11.9)	38.0 (11.4)	37.1 (11.5)	36.8 (11.5)	38.0 (11.4)
Male	244/311(78.0)	123/149(83.0)	121/162 (75.0)	214/270 (79.0)	30/41 (73.0)	153/190 (81.0)	123/149 (83.0)	30/41 (73.0)
Birth in a highly endemic area	104/311 (33.0)	56/149 (38.0)	48/162 (30.0)	95/270 (35.0)	9/41 (22.0)	65/190 (34.0)	56/149 (38.0)	9/41 (22.0)
<6 y of education	137/310 (44.0)	67/149 (45.0)	70/161 (43.0)	119/269 (44.0)	18/41 (44.0)	85/190 (45.0)	67/149 (45.0)	18/41 (44.0)
>4 h of daily contact w/cattle	181/310 (58.0)	91/149 (61.0)	90/161 (56.0)	161/269 (60.0)	20/41 (49.0)	111/190 (58.0)	91/149 (61.0)	20/41 (49.0)
>1 y of stay in the facility	283/309 (92.0)	140/149 (94.0)	143/160 (89.0)	245/268 (91.0)	38/41 (93.0)	178/190 (94.0)	140/149 (94.0)	38/41 (93.0)
High exposure activity	105/309 (34.0)	65/148 (44.0)**	40/161 (25.0)	100/268 (37.0)*	5/41 (12.0)	70/189 (37.0)	65/148 (44.0)**	5/41 (12.0)
Unpasteurized dairy products consumption	93/310 (30.0)	45/148 (30.0)	48/162 (30.0)	75/269 (28.0)*	18/41 (44.0)	63/189 (33.0)	45/148 (30.0)	18/41 (44.0)
BCG scar	265/301 (88.0)	126/143 (88.0)	139/158 (88.0)	231/261 (89.0)	34/40 (85.0)	160/183 (87.0)	126/143 (88.0)	34/40 (85.0)
Previous contact with TB cases	31/308 (10.0)	15/147 (10.0)	16/161 (10.0)	24/267 (9.0)	7/41 (17.0)	22/188 (12.0)	15/147 (10.0)	7/41 (17.0)

* p<.05;

** p<.001.

### TST+ and/or IGRA+ vs. TST− & IGRA− test result combination

Thirty seven percent (100/268) of the TST+ and/or IGRA+ and 12% (5/41) of the subjects with both negative tests results performed high exposure activities (p = 0.002) (Table 6). Multivariate analyses revealed association of this result with high exposure activities (OR 4.67 95% CI 1.62–13.47) (Table 5).

### TST+ & IGRA+ vs. TST− & IGRA− test result combination

Forty four percent (65/148) of the TST+ & IGRA+ subjects and 12% (5/41) of the subjects with both negative tests results performed high exposure activities (p<0.001). No other characteristic was found statistically different (Table 6). Multivariate analyses revealed association of this result with high exposure activities (OR 6.09 95% CI 2.04–18.23). (Table 5)

### Active tuberculosis

At the time of evaluation, 9.6% (30/311) of the subjects had had respiratory symptoms for more than two weeks. Of these, only two cases were bacteriologically confirmed with pulmonary TB. The prevalence of active disease among the study population was estimated to be of 643 cases/100,000 inhabitants.

The first case was identified in August 2010; the patient was a 48 year-old male, with type-2 diabetes mellitus, poorly controlled. When diagnosed, he worked as a salesman in a grocery store inside the facility, but he had worked as a milker in one of the cowsheds of this facility 12 years before. *M. bovis* was identified from sputa and showed a spoligotype (676 741 077 777 600) that was not present among the circulating strains in the cattle, and its lineage was different from those reported in the international electronic database. Following the guidelines of Mexico's National TB Control Program, (Modificación a la Norma Oficial Mexicana NOM-006-SSA2-1993, para la prevención y control de la tuberculosis en la atención primaria a la salud: Secretaria de Salud, Diario Oficial de la Federación, Mexico, 2000.), the patient received directly observed daily doses of two months isoniazid (H), rifampin (R) pyrazinamide (Z) and ethambutol IE) followed by a three times weekly six-month continuation phase of HR [2HRZE/6(HR)_3_] with a favorable clinical outcome. The second case was identified in April 2011; the patient was a 37 year-old woman, with rheumatoid arthritis under treatment with prednisone and methotrexate; she worked as a milker at the moment of diagnosis. *M. bovis* was isolated in her sputa and the spoligotype of the strain (SB0121) belonged to the BOVIS-1 linage and was identical to the isolates cultured from two animals from the same cowshed where she worked. The patient was treated same as above [2HRZE/6(HR)_3_] with a favorable clinical outcome.

## Discussion

In the present study we documented a very high prevalence of LTBI among DFW by two different methods (TST and IGRA). Despite this high baseline prevalence we were able to determine a higher probability of infection among those heavily exposed in indoor settings. We also identified cattle to human transmission with development of pulmonary disease, and in one of the cases, a matching spoligotype with one the circulating strains from the bovines of the same cowshed.

We hypothesize that the burden of LTBI in our study population is explained by endemic and repeated exposure to *M. bovis* infected cattle. The prevalence of LTBI found in the present study is higher than what has been informed in other studies performed in high-risk populations in Mexico, such as in border cities (57%) [18], migrant workers (26%) [19], and health care workers (43–64.5%) [20], [21], [22]. It is also higher than the prevalence estimates in open populations from several decades ago [23]. Compared with similar studies, the frequency of LTBI in our study is higher than the 45% prevalence reported among Mexican DFW during an outbreak of bovine tuberculosis in California [11].

Our estimations are well above the estimated regional prevalence of bovine TB of 1% [12]. We estimated the level of *M. bovis* infection amongst the 29,000 cattle at the facility by performing necropsies and mycobacterial culture on clinical material from animals that died during the study. *M. bovis* was culture confirmed in 154/1561 (9.8%) cattle that died, although the true estimate would likely have been higher if more necropsies and cultures had been performed. We consider that the true frequency of *M bovis* infection among cattle approaches 34.4% in this area, this figure resulting from macroscopic necropsy findings.

Although the present study was not designed to compare the prevalence of active TB with that occurring in surrounding areas, it is relevant and alarming that two cases of active pulmonary TB were found among DFW in such a short time frame, although in only one case were we able to link to the workplace via spoligotyping. Both cases were caused by *M. bovis*, which causes less than 4% of the cases of pulmonary tuberculosis in the general population and contributes with less than 2% of the overall TB burden. Therefore, we consider that most probably, the prevalence of active tuberculosis in this study population is much higher than the prevalence reported in Mexico (18.1 cases/100,000 habitants) [24]. This provides direct evidence of interspecies transmission, and supports previous reports in which close contact with animals or their tissues represents a risk for transmission. Working habits, poor ventilation, and animal crowding seem to be the most relevant risk factors associated with LTBI in our study, similar to the conditions usually found in *M. tuberculosis* disease. We did not identify consumption of unpasteurized dairy products as a risk factor, as others have found [25]. We consider that the apparent protective effect is spurious, since it is not biologically plausible and may have been subject to information bias. We believe that some of the workers may have deliberately denied the consumption of raw milk given that the consumption of the product was prohibited by the owner in some cowsheds. Other factors, such as history of prior contact with a TB case, or having lived in highly TB endemic areas were not statistically associated with LTBI after multivariate analysis. This may be explained by high prevalence of LTBI in the study population and the strength of the association with high exposure activities, which may have attenuated the impact of other factors.

Usage of two tests, TST and IGRA, allowed us to confirm that both tests are useful to identify occupational risks associated to LTBI. Analyses using different results combination allowed us to show consistency of the association. Odds ratio was found to be higher when a more specific combination was used (IGRA and TST positive compared with IGRA and TST negative results) [26].

### Study limitations

We recognize several limitations in the study. First, we did not sample all the facility personnel, because of the reluctance of some of the cowshed owners to allow access to their facilities. However, since we studied 46.6% of the total cowsheds and the cowsheds were similar in farming practices, we consider that our results may be representative of the problem in the study area. Secondly, since the tests we used are not specific for *M. bovis*, we may have detected infection caused by other species of *M. tuberculosis* complex [27]. It may be hypothesized that at least 30% to 35% of the cases could be attributed to *M. bovis* infection, since the prevalence of TST positivity in Mexico is around 40% in other settings [18], [19], [20]. Thirdly, given the fact that BCG vaccine is administered to all newborns in Mexico, we were unable to determine the proportion of TST reactivity due to BCG vaccination or to exposure to infected cattle. The interpretation of TST results in BCG vaccinated populations has been controversial. However, we consider that TST was useful in our study to identify individuals who had been exposed to infected cattle. TST reactivity associated with neonatal BCG vaccination has been shown to wane after several years [28]. Furthermore, we have previously documented that the TST used together with a standardized questionnaire eliciting information about risk factors for exposure to infectious tuberculosis can identify individuals who have been exposed to an active pulmonary tuberculosis case in an area with high BCG vaccination coverage [26]. Therefore, we believe that the difference in the positivity rates between TST and IGRA cannot be completely attributed to BCG vaccination, but may also reflect the inaccurate performance of IGRA in high tuberculosis burden settings [29].

In conclusion, our findings provide evidence of occupational risk in a dairy production and livestock facility in Mexico and may be representative of settings in which close contact of humans and infected animals occurs. Based on this evidence, it is recommended to establish more stringent prevention and control measures of bovine tuberculosis including protection to workers, especially those who are exposed in closed environments.

## References

[1] Hlavsa MC, Moonan PK, Cowan LS, Navin TR, Kammerer JS, et al (2008) Human tuberculosis due to Mycobacterium bovis in the United States, 1995–2005. Clin Infect Dis 47: 168–175. 18532886 10.1086/589240

[2] Michel AL, Muller B, van Helden PD (2010) Mycobacterium bovis at the animal-human interface: a problem, or not? Vet Microbiol 140: 371–381. 19773134 10.1016/j.vetmic.2009.08.029

[3] Torgerson P, Torgerson D (2008) Does risk to humans justify high cost of fighting bovine TB? Nature 455: 1029. 10.1038/4551029a 18948927

[4] LoBue PA, Betacourt W, Peter C, Moser KS (2003) Epidemiology of Mycobacterium bovis disease in San Diego County, 1994–2000. Int J Tuberc Lung Dis 7: 180–185. 12588020

[5] Human tuberculosis caused by Mycobacterium bovis–New York City, 2001–2004. MMWR Morb Mortal Wkly Rep 54: 605–608. 15973241

[6] Rodwell TC, Moore M, Moser KS, Brodine SK, Strathdee SA (2008) Tuberculosis from Mycobacterium bovis in binational communities, United States. Emerg Infect Dis 14: 909–916. 18507901 10.3201/eid1406.071485 PMC2600278

[7] Cousins DV, Dawson DJ (1999) Tuberculosis due to Mycobacterium bovis in the Australian population: cases recorded during 1970–1994. Int J Tuberc Lung Dis 3: 715–721. 10460105

[8] Robinson P, Morris D, Antic R (1988) Mycobacterium bovis as an occupational hazard in abattoir workers. Aust N Z J Med 18: 701–703. 3072951 10.1111/j.1445-5994.1988.tb00156.x

[9] Fanning A, Edwards S, Hauer G (1991) Mycobacterium bovis infection in humans exposed to elk in Alberta. Can Dis Wkly Rep 17: 239–240, 243. 1841004

[10] Moda G, Daborn CJ, Grange JM, Cosivi O (1996) The zoonotic importance of Mycobacterium bovis. Tuber Lung Dis 77: 103–108. 8762842 10.1016/s0962-8479(96)90022-2

[11] Winthrop KL, Scott J, Brown D, Jay MT, Rios R, et al (2005) Investigation of human contacts: a Mycobacterium bovis outbreak among cattle at a California dairy. Int J Tuberc Lung Dis 9: 809–813. 16013779

[12] Cosivi O, Grange JM, Daborn CJ, Raviglione MC, Fujikura T, et al (1998) Zoonotic tuberculosis due to Mycobacterium bovis in developing countries. Emerg Infect Dis 4: 59–70. 9452399 10.3201/eid0401.980108 PMC2627667

[13] Zoonotic tuberculosis (Mycobacterium bovis): memorandum from a WHO meeting (with the participation of FAO). Bull World Health Organ 72: 851–857. 7867130 PMC2486730

[14] Liss GM, Wong L, Kittle DC, Simor A, Naus M, et al (1994) Occupational exposure to Mycobacterium bovis infection in deer and elk in Ontario. Can J Public Health 85: 326–329. 7804937

[15] Mazurek GH, Jereb J, Vernon A, LoBue P, Goldberg S, et al (2010) Updated guidelines for using Interferon Gamma Release Assays to detect Mycobacterium tuberculosis infection - United States, 2010. MMWR Recomm Rep 59: 1–25. 20577159

[16] Denkinger CM, Dheda K, Pai M (2011) Guidelines on interferon-gamma release assays for tuberculosis infection: concordance, discordance or confusion? Clin Microbiol Infect 17: 806–814. 21682801 10.1111/j.1469-0691.2011.03555.x

[17] Kamerbeek J, Schouls L, Kolk A, van Agterveld M, van Soolingen D, et al (1997) Simultaneous detection and strain differentiation of Mycobacterium tuberculosis for diagnosis and epidemiology. J Clin Microbiol 35: 907–914. 9157152 10.1128/jcm.35.4.907-914.1997 PMC229700

[18] Laniado-Laborin R, Cabrales-Vargas N, Lopez-Espinoza G, Lepe-Zuniga JL, Quinonez-Moreno S, et al (1998) [Prevalence of tuberculosis infection in students in the city of Tijuana, Mexico]. Salud Publica Mex 40: 47–52. 9580502

[19] Trape-Cardoso M, Subaran S, Bracker A, Sapiain E, Gould B (2008) Latent tuberculosis among Latino migrant farmworkers in Connecticut. Conn Med 72: 405–409. 18763668

[20] Ostrosky-Zeichner L, Rangel-Frausto MS, Garcia-Romero E, Vazquez A, Ibarra MJ, et al (2000) [Tuberculosis in health personnel: importance of surveillance and control programs]. Salud Publica Mex 42: 48–52. 10743399

[21] Molina-Gamboa JD, Ponce-de-Leon-Rosales S, Rivera-Morales I, Romero C, Baez R, et al (1994) Evaluation of the sensitivity of RT-23 purified protein derivative for determining tuberculin reactivity in a group of health care workers. Clin Infect Dis 19: 784–786. 7803652 10.1093/clinids/19.4.784

[22] Garcia-Garcia ML, Jimenez-Corona A, Jimenez-Corona ME, Ferreyra-Reyes L, Martinez K, et al (2001) Factors associated with tuberculin reactivity in two general hospitals in Mexico. Infect Control Hosp Epidemiol 22: 88–93. 11232884 10.1086/501869

[23] Cardenas-Ayala VM, Bernal-Perez J, Cabrera-Coello L, Stetler HC, Pineda-Salgado J, et al (1989) [Tuberculosis surveys in Guerrero and new estimates of the magnitude of tuberculosis infection in Mexico]. Salud Publica Mex 31: 73–81. 2711258

[24] WHO (2010) Tb country profile: Mexico. Available: http://www.who.int/tb/country/data/profiles/en/

[25] Besser RE, Pakiz B, Schulte JM, Alvarado S, Zell ER, et al (2001) Risk factors for positive mantoux tuberculin skin tests in children in San Diego, California: evidence for boosting and possible foodborne transmission. Pediatrics 108: 305–310. 11483792 10.1542/peds.108.2.305

[26] Garcia-Sancho FM, Garcia-Garcia L, Jimenez-Corona ME, Palacios-Martinez M, Ferreyra-Reyes LD, et al (2006) Is tuberculin skin testing useful to diagnose latent tuberculosis in BCG-vaccinated children? Int J Epidemiol 35: 1447–1454. 17008360 10.1093/ije/dyl213

[27] Pai M, Riley LW, Colford JM Jr (2004) Interferon-gamma assays in the immunodiagnosis of tuberculosis: a systematic review. Lancet Infect Dis 4: 761–776. 15567126 10.1016/S1473-3099(04)01206-X

[28] Kroger L, Katila ML, Korppi M, Brander E, Pietikainen M (1992) Rapid decrease in tuberculin skin test reactivity at preschool age after newborn vaccination. Acta Paediatr 81: 678–681. 1421907 10.1111/j.1651-2227.1992.tb12332.x

[29] Dheda K, van Zyl Smit R, Badri M, Pai M (2009) T-cell interferon-gamma release assays for the rapid immunodiagnosis of tuberculosis: clinical utility in high-burden vs. low-burden settings. Curr Opin Pulm Med 15: 188–200. 19387262 10.1097/MCP.0b013e32832a0adc

